# DeepDelta: predicting ADMET improvements of molecular derivatives with deep learning

**DOI:** 10.1186/s13321-023-00769-x

**Published:** 2023-10-27

**Authors:** Zachary Fralish, Ashley Chen, Paul Skaluba, Daniel Reker

**Affiliations:** 1 Department of Biomedical Engineering, Duke University, Durham, NC 27708 USA; 2 Department of Computer Science, Duke University, Durham, NC 27708 USA

**Keywords:** Machine learning, Drug design, Molecular optimization, Neural network, ADMET, Drug development

## Abstract

Established molecular machine learning models process individual molecules as inputs to predict their biological, chemical, or physical properties. However, such algorithms require large datasets and have not been optimized to predict property differences between molecules, limiting their ability to learn from smaller datasets and to directly compare the anticipated properties of two molecules. Many drug and material development tasks would benefit from an algorithm that can directly compare two molecules to guide molecular optimization and prioritization, especially for tasks with limited available data. Here, we develop DeepDelta, a pairwise deep learning approach that processes two molecules simultaneously and learns to predict property differences between two molecules from small datasets. On 10 ADMET benchmark tasks, our DeepDelta approach significantly outperforms two established molecular machine learning algorithms, the directed message passing neural network (D-MPNN) ChemProp and Random Forest using radial fingerprints, for 70% of benchmarks in terms of Pearson’s r, 60% of benchmarks in terms of mean absolute error (MAE), and all external test sets for both Pearson’s r and MAE. We further analyze our performance and find that DeepDelta is particularly outperforming established approaches at predicting large differences in molecular properties and can perform scaffold hopping. Furthermore, we derive mathematically fundamental computational tests of our models based on mathematical invariants and show that compliance to these tests correlates with overall model performance — providing an innovative, unsupervised, and easily computable measure of expected model performance and applicability. Taken together, DeepDelta provides an accurate approach to predict molecular property differences by directly training on molecular pairs and their property differences to further support fidelity and transparency in molecular optimization for drug development and the chemical sciences.

**Supplementary Information:**

The online version contains supplementary material available at 10.1186/s13321-023-00769-x.

## Introduction

**Fig. 1:**
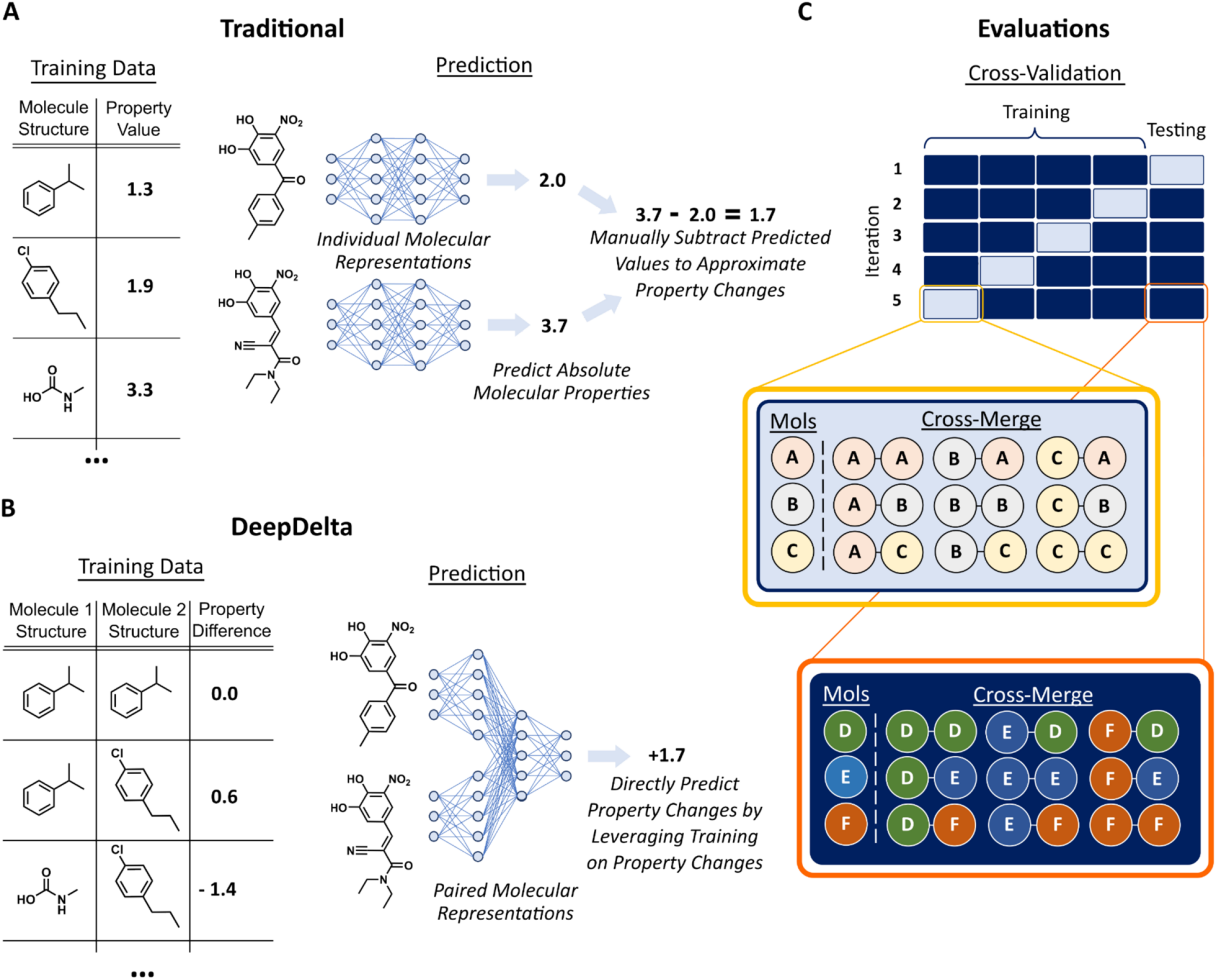
Traditional and pairwise architectures. **A** Traditional molecular machine learning models take singular molecular inputs and predict absolute properties of molecules. Predicted property differences can be calculated by subtracting predicted values for two molecules. **B** Pairwise models train on differences in properties from pairs of molecules to directly predict property changes of molecular derivatizations. **C** Molecules are cross-merged to create pairs only after cross-validation splits to prevent the risk of data leakage during model evaluation. Therefore, every molecule in the dataset can only occur in pairs in the training or testing data, but not both

Drug design requires a balancing act between optimizing the on-target potency of a drug lead and maintaining an appropriate absorption, distribution, metabolism, excretion, and toxicity (ADMET) profile [1]. To this end, lead series are extensively characterized experimentally to compare properties of compounds and identify the most promising candidates. Unfortunately, such characterizations are laborious and expensive and can include complex in vivo experiments [2]. Therefore, many such characterizations are often restricted to only a small set of candidate compounds, which causes an incomplete understanding of the structure–activity relationship and risks the premature elimination of candidates with potentially beneficial properties. To accelerate and economize the characterization of compound properties while enabling the evaluation of larger sets of candidates, computational approaches are increasingly deployed in pharmaceutical development [1]. Molecular machine learning algorithms learn from large historic data to directly predict the absolute property values of a molecule from its chemical structure (Fig. 1a) and are now commonly utilized in both industry [3, 4] and academia [5, 6] to triage experimental testing. Such machine learning workflows are becoming increasingly accurate due to expanding availability of training data, growing computational power, and improvements in predictive algorithms [5]. However, molecular machine learning algorithms are not yet optimized to directly compare the properties of two molecular structures to inform compound optimization and enable lead series prioritization through direct contrasting of expected molecular properties.

There are several related, powerful approaches to predict property differences between two molecules, but they have important shortcomings that limit their broad practical deployment. For example, one of the most powerful approaches to predict property differences between two molecules is Free Energy Perturbations (FEP), with promising results in ab initio molecular optimization. However, FEP calculations are prohibitively complex and resource intensive, which hinders their broad deployment [7]. Although “DeltaDelta” neural networks have emerged to predict binding affinity differences for two molecules more rapidly than previous algorithms, their use of protein–ligand complexes as input requires costly structural biology [8, 9]. Conversely, Matched Molecular Pair (MMP) analysis allows the rapid anticipation of property differences but can only predict differences between close molecular derivatives, is limited to common molecular derivations, and can fail to account for important chemical context [10].

Here, we evaluate the potential of two state-of-the-art molecular machine learning algorithms, classic Random Forest models [11] and the message passing neural network ChemProp [12], to predict ADMET property differences between two molecular structures. We chose Random Forest to represent classical machine learning methods given its robust performance for molecular machine learning tasks [13–16] and chose ChemProp to represent deep learning methods as it leverages a hybrid representation of convolutions centered on bonds and exhibits strong predictive power for a range of molecular property benchmark datasets [12]. Both methods show mediocre resolution to correctly predict property differences, limiting their utility for molecular optimization tasks. Motivated by this shortcoming, we propose DeepDelta, which directly learns property differences for pairs of molecules (Fig. 1b). DeepDelta shows significantly improved performance in most (82% in terms of Pearson’s r and 73% in terms of MAE) of our benchmarks that include cross-validation and external test set experiments. We analyze additional properties of DeepDelta from first mathematical principles, which enables us to derive accurate and rapidly calculable confidence measures that are predictive of the model’s performance. In contrast to existing molecular comparison approaches such as FEP and MMP, our DeepDelta approach can rapidly predict property differences between millions of chemically unrelated molecular pairs while accounting for molecular context without requiring complex ab initio calculations or protein–ligand complexes. Taken together, we believe that DeepDelta and extensions thereof will enable more accurate and holistic prioritization of drug lead series and thereby enable computation to support drug development more productively.

## Methods

### Datasets

We extracted 10 publicly available datasets of various ADMET properties [18–26] primarily from the Therapeutics Data Commons [27] (Table 1). Invalid SMILES were removed from all datasets except for “Hemolytic Toxicity”, in which incorrectly notated amine groups were manually corrected based on original literature sources. Datapoints originally annotated as “>” or “<” instead of “=” were removed. We log-transformed all datasets except for the “FreeSolv dataset”, in which negative values prohibit log-transformation. For the renal clearance dataset, we incremented all annotated values by one to avoid values of zero during log-transformation. Distributions of transformed values for all datasets are shown in Additional file 1: Fig. S1.

**Table 1:** Benchmarking datasets

Index	Property	Size	Units	References
1	Fraction Unbound, Brain	253	Log(f_u,brain_)	[23]
2	Renal Clearance	636	Log(CLr)	[25]
3	Free Solvation	642	Experimental Hydration Free Energy in Water	[20]
4	Microsomal Clearance	731	Log(mL/min/kg cleared)	[24]
5	Hemolytic Toxicity	828	Log(HD_50_)	[18]
6	Hepatic Clearance	881	Log(mL/min/kg cleared)	[24]
7	Caco2	910	Log(Papp)	[19]
8	Aqueous Solubility	1128	LogS	[26]
9	Volume of Distribution at Steady State	1130	Log(Body/Blood Concentration in L/kg)	[21]
10	Half-Life	1321	Log(Half-Life in Hours)	[22]

Description of the 10 benchmarking datasets

External test sets were collected from primary literature sources [28, 29] using the ChEMBL database [30] to identify suitable publications. All invalid SMILES were removed. All datapoints annotated as “>” or “<” instead of “=” were removed. Datapoints in the external datasets that were also present in the training data were identified and removed based on Tanimoto similarity using Morgan circular fingerprints (radius 2, 2048 bits, RDKit version 2022.09.5 [31], threshold of 1.0 to remove identical molecules). Datapoint values in the external test sets were log-transformed to match training data while removing any datapoints with an initial value of 0.

### Model architecture and implementation

To develop DeepDelta, we used the same underlying D-MPNN architecture as ChemProp given its efficient computation and its competitive performance on molecular data [12]. Furthermore, by building on this architecture, our results become easily comparable to the ChemProp implementation and allow us to directly quantify the benefit of our molecular pairing approach. Two molecules form an input pair for DeepDelta, while ChemProp processes a single molecule to predict absolute property values that are then subtracted to calculate property differences between two molecules. By training on input pairs and their property differences, DeepDelta directly learns and predicts property changes instead of requiring manual subtraction of predicted properties to approximate property changes. For ChemProp and DeepDelta, molecules were described using atom and bond features as previously implemented [12]. In short, molecular graphs are converted into a latent representation by passing through a D-MPNN. For DeepDelta, this is done separately for each molecule and the latent representations of both molecules are subsequently concatenated. The concatenated embedding is then passed through a second neural network for property prediction that consists of linear feed forward layers [32]. Both deep learning models were implemented for regression with default parameters and aggregation = ‘sum’ using the PyTorch deep learning framework. For the traditional ChemProp implementation, number_of_molecules = 1 while for DeepDelta number_of_molecules = 2 to allow for processing of multiple inputs [32]. We optimized the number of epochs for every model and set epochs = 5 for DeepDelta and epochs = 50 for ChemProp (Additional file 1: Fig. S2).

For Random Forest and Light Gradient Boosting Machine (LightGBM, Microsoft) models, molecules were described using radial chemical fingerprints (Morgan circular fingerprint, radius 2, 2048 bits, rdkit.org). The Random Forest regression machine learning models with 500 trees were implemented with default parameters in scikit-learn. The LightGBM was implemented with a subsample frequency of 0.1 to further improve running time on large datasets (LGBMsub) and otherwise default parameters, except for in the “Fraction Unbound, Brain” dataset, where we used min_child_samples = 5 due to the small size of the original dataset. For traditional implementations of Random Forest and LGBMsub, each molecule was processed individually (i.e., predictions are made solely based on the fingerprint of a single molecule), and property differences are calculated by making two separate predictions (one for each molecule) and these predictions are subsequently subtracted to calculate property differences between two molecules. For the delta version of LGBMsub, fingerprints for paired molecules were concatenated to form paired molecular representations to directly train on and predict property changes. LightGBM models were implemented to evaluate pairwise methods applied to classic tree-based machine learning methods due to LGBMsub’s increased efficiency in handling large datasets compared to other tree-based methods [33].

### Model evaluation

Models were evaluated using 5 × 10-fold cross-validation (sklearn), and performance was measured using Pearson’s r, MAE, and root mean squared error (RMSE). To prevent data leakage, training data was first split into train and test sets during cross-validation prior to cross-merging to create molecule pairings (Fig. 1c); i.e., every molecule will only be present in pairs made from either the training or the test set but not both. Through this method, all possible pairs within a set are made. Additionally, the order of molecules matters, preserving both the magnitude and direction of property changes. Plots of cross-validation results were made with matplotlib from cross-validation splits with a random state = 1. MMP analysis [17] was implemented in KNIME using nodes from the RDKit and Vernalis community extensions. SMILES were preprocessed by de-salting, removing explicitly defined stereocenters and double bond geometries, canonicalizing, and filtering duplicates. Following fragmentation, matched molecular pairs were identified and grouped together using the canonical SMILES. Scaffold analysis and comparisons of Tanimoto similarity, delta values, and predictive errors were made with cross-validation splits with a random state = 1 and plotted with matplotlib. Analysis of additional properties of DeepDelta were made with cross-validation splits with a random state = 1. Paired *t*-tests were performed for comparison of the five repeats of our ten-fold cross-validation and the Kolmogorov–Smirnov test was performed to assess normality of all distributions prior to comparisons with paired *t*-tests. Overall comparisons of performance across benchmarks were assessed using the non-parametric Wilcoxon signed-rank test. Code and data for all these calculations can be found at https://github.com/RekerLab/DeepDelta.

## Results

### Performance of established approaches

**Fig. 2:**
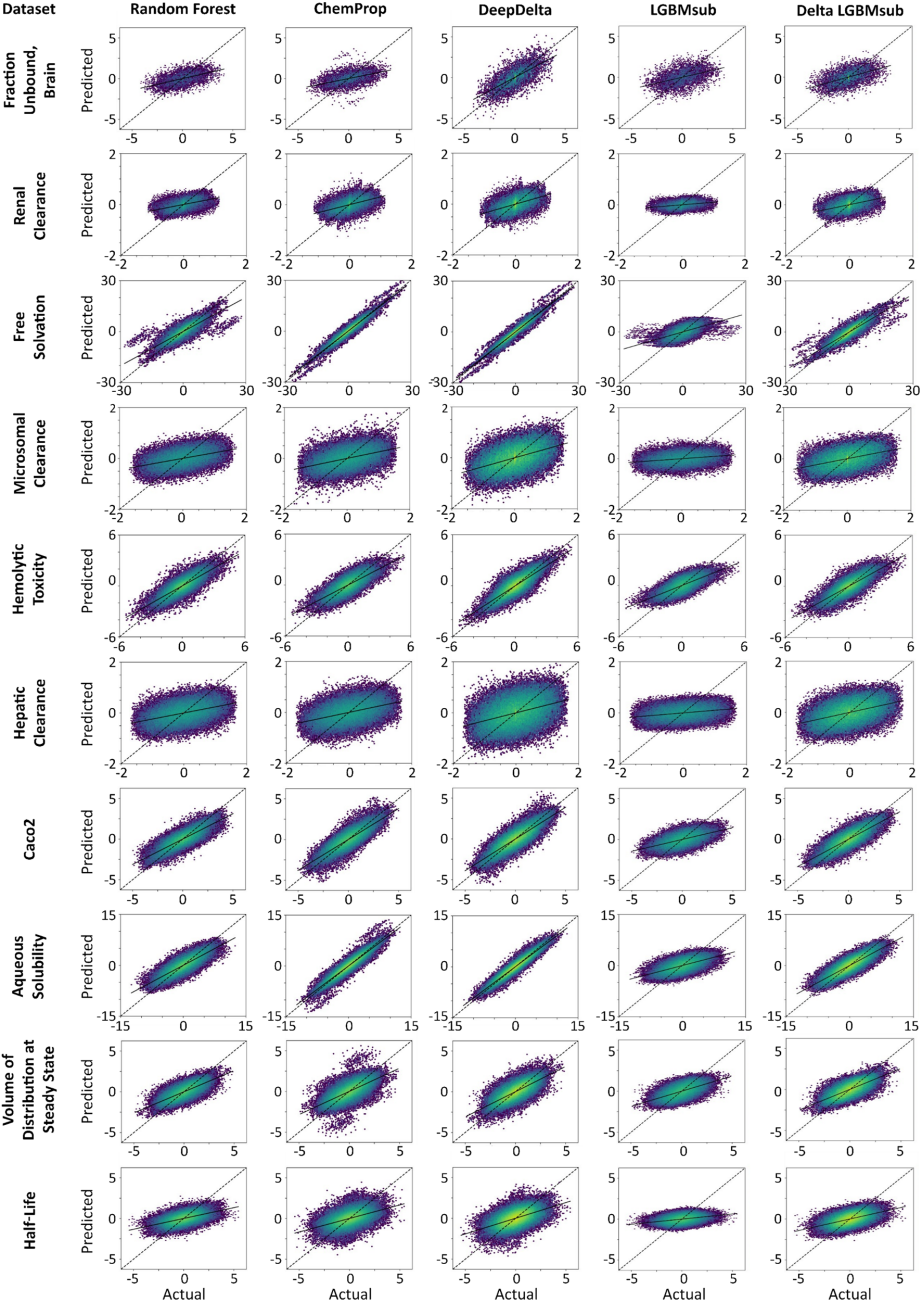
Cross-validation results across benchmark datasets. Correlation plots for Random Forest, ChemProp, DeepDelta, LGBMsub, and Delta LGBMsub following 5 × 10-fold cross-validation. Datasets are sorted by size from smallest (top) to largest (bottom). Coloring is based on data density with the most densely populated regions shown in yellow, least dense regions in blue, and linear interpolation between these groups

We first investigated whether established classical machine learning (Random Forest using Morgan circular fingerprints) [11] and graph-based deep learning (ChemProp) [12] algorithms could be used to predict differences in ADMET properties between two molecular structures. For this, we split all our benchmark datasets randomly into training and testing sets following a cross-validation strategy. The models (Random Forest or ChemProp) were then trained on the training folds and used to predict the properties of the molecules in the testing fold. Instead of directly evaluating the predicted property values of the test set molecules against the annotated ground truth, as is usually done, we evaluated the ability of our models to predict relative property differences between all possible pairs of molecules in the test set by subtracting their predicted property values and comparing these differences to the subtracted ground truth property values (Fig. 1a). In other words, absolute properties of individual molecules were predicted using individual molecular representations, and the predicted values were then subsequently subtracted to approximate molecular differences, meaning the models are not directly predicting property differences. We found overall mediocre performance of these established machine learning algorithms to predict property differences with median Pearson’s r values across all benchmarking datasets of 0.60 for ChemProp and 0.63 for the Random Forest models (Fig. 2 and Table 2). This limited performance illuminates an opportunity for novel machine learning approaches tailored to predict property differences between molecules to improve our predictive power and resolution for molecular optimizations. Of note, we also explored the option of using MMP on these benchmark datasets, but standard MMP implementations [17] can only make predictions for 0.6% of the molecular pairs in our data, highlighting the necessity of a more broadly applicable approach.

**Table 2:** Evaluations of 5 × 10-fold cross-validation of random forest, ChemProp, DeepDelta, LGBMsub, and Delta LGBMsub

Dataset	Pearson’s r					Mean absolute error					Root mean squared error				
	RF	ChemProp	DeepDelta	LGBMsub	Delta LGBMsub	RF	ChemProp	DeepDelta	LGBMsub	Delta LGBMsub	RF	ChemProp	DeepDelta	LGBMsub	Delta LGBMsub
Fraction Unbound, Brain	0.535 ± 0.008	0.483 ± 0.023	**0.701 ± 0.013**	0.47 ± 0.041	0.53 ± 0.015	0.713 ± 0.008	0.740 ± 0.01	**0.642 ± 0.014**	1.003 ± 0.008	0.726 ± 0.011	0.928 ± 0.01	0.965 ± 0.019	**0.830 ± 0.023**	1.311 ± 0.014	0.939 ± 0.014
Renal Clearance	**0.461 ± 0.01**	**0.478 ± 0.013**	**0.465 ± 0.023**	0.283 ± 0.016	0.443 ± 0.007	**0.256 ± 0.002**	0.262 ± 0.004	0.269 ± 0.006	0.307 ± 0.003	0.265 ± 0.002	**0.337 ± 0.002**	**0.339 ± 0.005**	0.350 ± 0.008	0.396 ± 0.004	0.344 ± 0.002
Free Solvation	0.843 ± 0.005	0.967 ± 0.0	**0.971 ± 0.001**	0.589 ± 0.007	0.898 ± 0.003	1.874 ± 0.028	0.937 ± 0.006	**0.806 ± 0.003**	4.23 ± 0.058	1.656 ± 0.027	2.914 ± 0.045	1.372 ± 0.009	**1.290 ± 0.023**	5.472 ± 0.064	2.415 ± 0.037
Microsomal Clearance	**0.444 ± 0.002**	0.451 ± 0.011	**0.468 ± 0.007**	0.273 ± 0.011	0.42 ± 0.01	**0.434 ± 0.002**	**0.436 ± 0.003**	0.444 ± 0.002	0.509 ± 0.004	0.443 ± 0.002	**0.544 ± 0.001**	**0.546 ± 0.005**	0.557 ± 0.002	0.638 ± 0.005	0.556 ± 0.003
Hemolytic Toxicity	0.821 ± 0.002	0.778 ± 0.003	**0.842 ± 0.002**	0.706 ± 0.007	0.816 ± 0.003	0.506 ± 0.002	0.566 ± 0.006	**0.487 ± 0.004**	0.897 ± 0.008	0.516 ± 0.004	0.663 ± 0.005	0.729 ± 0.007	**0.635 ± 0.006**	1.127 ± 0.01	0.672 ± 0.005
Hepatic Clearance	**0.438 ± 0.004**	**0.431 ± 0.005**	0.392 ± 0.007	0.28 ± 0.013	0.424 ± 0.013	**0.45 ± 0.002**	0.455 ± 0.002	0.494 ± 0.003	0.529 ± 0.006	0.46 ± 0.005	**0.564 ± 0.002**	0.570 ± 0.002	0.622 ± 0.004	0.663 ± 0.008	0.575 ± 0.005
Caco2	0.829 ± 0.006	0.851 ± 0.005	**0.853 ± 0.005**	0.565 ± 0.013	0.829 ± 0.004	0.472 ± 0.006	0.451 ± 0.006	**0.444 ± 0.006**	0.82 ± 0.012	0.473 ± 0.005	0.614 ± 0.01	**0.575 ± 0.007**	**0.572 ± 0.008**	1.036 ± 0.014	0.61 ± 0.007
Aqueous Solubility	0.837 ± 0.003	0.951 ± 0.001	**0.957 ± 0.001**	0.596 ± 0.006	0.852 ± 0.003	1.237 ± 0.003	0.687 ± 0.005	**0.644 ± 0.006**	2.184 ± 0.029	1.197 ± 0.004	1.623 ± 0.011	0.915 ± 0.008	**0.859 ± 0.012**	2.783 ± 0.038	1.562 ± 0.011
Volume of Distribution at Steady State	0.728 ± 0.003	0.697 ± 0.003	**0.746 ± 0.005**	0.578 ± 0.006	0.719 ± 0.003	0.483 ± 0.003	0.505 ± 0.004	**0.470 ± 0.004**	0.772 ± 0.006	0.493 ± 0.003	0.632 ± 0.003	0.670 ± 0.004	**0.618 ± 0.006**	0.969 ± 0.008	0.64 ± 0.003
Half-life	**0.529 ± 0.006**	0.508 ± 0.008	**0.534 ± 0.004**	0.330 ± 0.007	0.514 ± 0.003	**0.586 ± 0.003**	0.600 ± 0.003	0.597 ± 0.003	0.662 ± 0.003	0.594 ± 0.005	**0.762 ± 0.003**	0.784 ± 0.004	0.778 ± 0.004	0.849 ± 0.003	0.771 ± 0.002

Average and standard deviation of Pearson’s r, MAE, and RMSE are presented for all 5 models. Best performance per dataset (*p* < 0.05) is bolded

### DeepDelta improves performance

We hypothesized that a neural network specifically trained to predict property differences could potentially outperform established machine learning models on this task. To test this, we generated a new machine learning task in which every datapoint is composed of a pair of molecules and the objective variable is the difference in their properties (Fig. 1b). This data serves as input to a deep learning model that accepts two molecules as inputs and predicts the property difference between these molecules. This new approach, retrospectively tested on all our benchmark datasets using the same cross-validation scheme, significantly outperformed Random Forest and ChemProp on the level of the individual benchmarks (*p* = 0.006) and achieved a promising, higher median Pearson’s r of 0.72 (Table 2). Through the combinatorial expansion of training data resulting from pairing, DeepDelta also converged more rapidly while implementations of deep models that process a single molecule to predict absolute property values typically require training for multiple epochs to converge when used on small datasets (Additional file 1: Fig. S2). The rapid convergence and improved performance of the DeepDelta approach over the standard implementation of providing individual molecules to ChemProp highlights how this method can allow smaller datasets (< 1500 datapoints) to be more effectively processed by deep learning methods that are more data hungry.

When comparing the performance of DeepDelta to ChemProp or Random Forest models on the level of individual benchmarks (Fig. 2), DeepDelta performed similar or better in 90% of the benchmarks when considering Pearson’s r. DeepDelta showed the most pronounced improvement for the “Fraction Unbound, Brain” dataset with improvements of at least 0.17 according to Pearson’s r and an MAE reduction of at least 0.07 compared to other models. While improvements were less pronounced in other datasets, DeepDelta still statistically outcompeted ChemProp in 70% of datasets (*p* < 0.05) and Random Forest in 70% of datasets (*p* < 0.05) for Pearson’s r with no significant change compared to each control model for two of the remaining datasets. DeepDelta exhibited a moderate but significant average improvement in Pearson’s r across all datasets of 0.04 (*p* = 0.006), with a maximum improvement of 0.22. DeepDelta also outcompeted 60% of the benchmarks in terms of MAE (Table 2) and exhibited a small, but significant average improvement in MAE across all datasets of 0.13 (*p* = 0.04), with a maximum improvement of 1.063. It is worth noting that all applied models showed poor performance (Pearson’s r < 0.5) on the three datasets related to clearance and only moderate predictivity for half-life, possibly driven by the complexity of predicting clearance from the molecular structure alone when provided with limited data that does not fully capture all the different elimination pathways for a specific tissue. In particular, “Hepatic Clearance” is the only benchmarking dataset where the DeepDelta approach is significantly outperformed by the other models in terms of Pearson’s r. In the future, we expect increasing amounts of data for specific elimination pathways to enable better predictions for all models for such tasks and to particularly benefit DeepDelta to more accurately capture differences in elimination between two structures. Already, the competitive performance of our pairing approach compared to established approaches highlights the ability of DeepDelta to improve performance of machine learning for current datasets of ADMET properties with large potential for further development.

### Tree-based delta approach

To further evaluate whether our new paired machine learning task could also be solved by classical tree-based machine learning methods, we implemented Microsoft’s Light Gradient Boosting Machine (LightGBM) that we parametrized to subsample the training data for more efficient training on large datasets (LGBMsub). Analogously to the training of DeepDelta, we provided the Delta LGBMsub models with a representation of both molecules by concatenating Morgan circular fingerprints of the two molecules and trained them on property differences between the two molecules. Compared to the performance of the traditional LGBMsub models (i.e., trained on individual molecules and calculating predicted differences by subtracting predictions analogously to Fig. 1a), the paired Delta LGBMsub models showed significant improvement in Pearson’s r, MAE, and RMSE across all benchmark datasets during retrospective cross-validations (Fig. 2, Table 2). These data suggest that the paired machine learning task can improve the performance of classical machine learning algorithms when predicting property differences, but apparently to a lesser extent than the deep learning approach, as DeepDelta outperformed the paired Delta LGBMsub approach in all but one benchmark in terms of Pearson’s r (*p* < 0.05) and 60% of benchmarks in terms of MAE. The difference between the traditional LGBMsub and the paired Delta LGBMsub could be further reduced through parameter optimization and by reducing subsampling (Additional file 1: Table S1). These results indicate that the molecular pairing approach can also be beneficial to tree-based architectures but appears most promising for deep neural networks where the combinatorial data explosion leads to significant performance improvements during cross-validation.

### Performance on external data

**Fig. 3:**
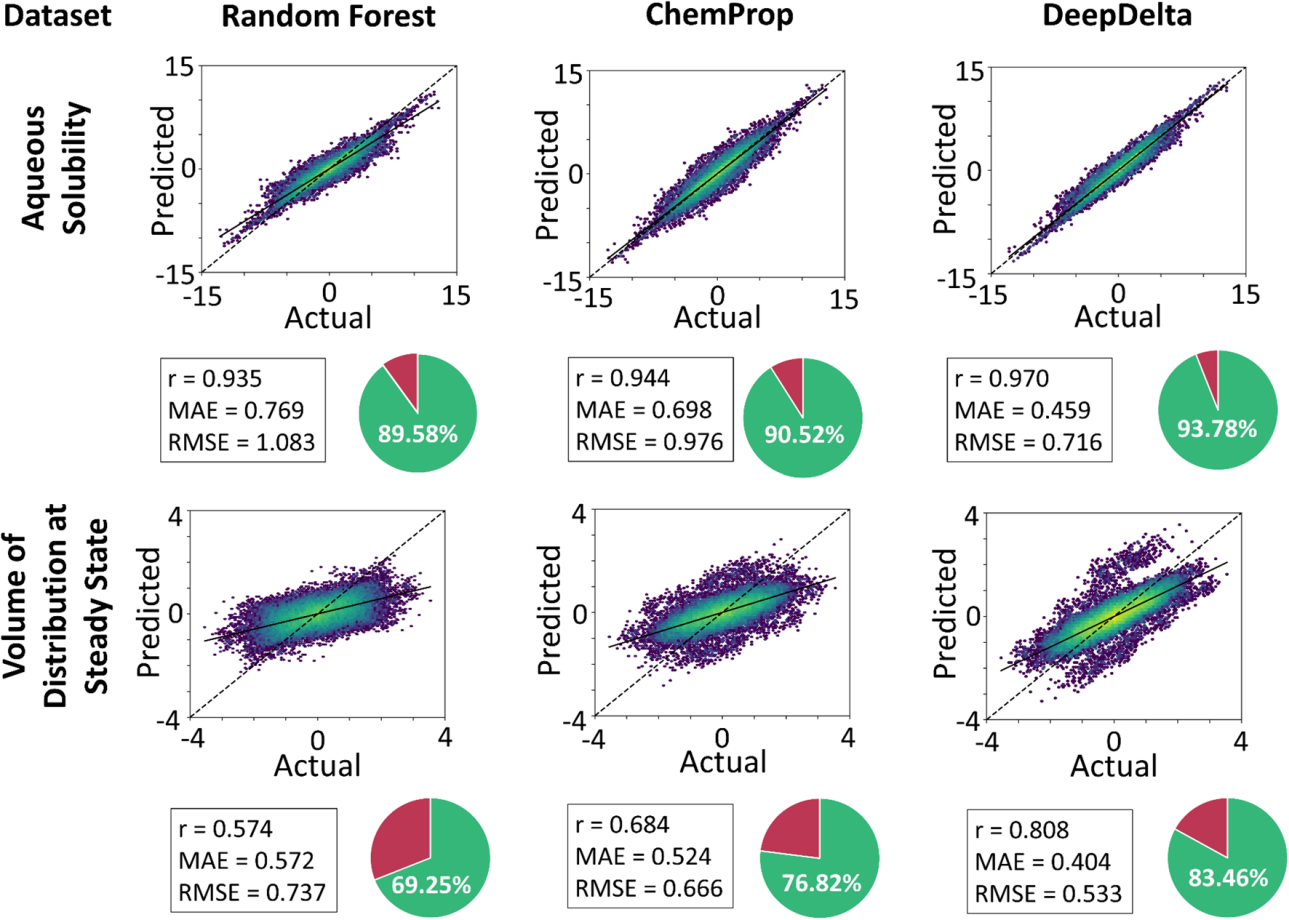
Model performance on external datasets. Correlation plots, Pearson’s r values, MAE, RMSE, and total percent of predictions correctly indicating a positive or negative change from the starting molecule (pie charts) for Random Forest, ChemProp, and DeepDelta models on cross-merged external test sets. Aqueous solubility is in units of logS and volume of distribution at steady state is in units of log(body/blood concentration in L/kg). Coloring is based on data density with the most densely populated regions shown in yellow, medium density shown in green, and least dense regions in blue and linear interpolation between these groups

We next investigated the generalizability of our new DeepDelta models by testing their performance on external test data. We sought external data for our three largest datasets, however, publicly available external datasets of appropriate size for “Half-life” overlapped with the training set or were derived through a different methodology (i.e., in vitro/in vivo animal assays instead of human clinical data). Therefore, we focused our external evaluation on “Solubility” and “Volume of Distribution at Steady State”. When training our models on our complete training data for these benchmarks and predicting pairs made exclusively from compounds in the external validation test sets, DeepDelta outperformed both Random Forest and ChemProp in all cases in terms of Pearson’s r, MAE, and RMSE and in accuracy, defined as the percent of predictions correctly predicting a positive or negative property change (Fig. 3). Similarly, the paired LGBMsub approach showed improvements across all metrics on the external test sets compared to the traditional LGBMsub (Additional file 1: Fig. S3) but did not outperform DeepDelta. Together, these results highlight the potential for DeepDelta to support molecular optimization by accurately predicting effects on ADMET properties arising from chemical modifications even for compound pairs that originate from other datasets, suggesting that DeepDelta can effectively generalize and predict property differences between molecules outside of the training data.

### Mathematical invariants

Apart from being able to make accurate predictions for property differences between two molecules, the pairing approach will also result in additional properties of our machine learning models. Specifically, an accurate DeepDelta model should capture the following three properties: predict zero property differences when provided the exact same molecule for both inputs,

1 $${\text{DeepDelta}}\left( {x,x} \right) = 0$$

predict the inverse of the original prediction when swapping the input molecules,

2 $${\text{DeepDelta}}\left( {x,y} \right) = - \,{\text{DeepDelta}}\left( {y,x} \right)$$

and preserve additivity for predicted differences between three molecules,

3 $${\text{DeepDelta}}\left( {x,y} \right) + {\text{DeepDelta}}\left( {y,z} \right) = {\text{DeepDelta}}\left( {x,z} \right)$$

We analyzed our data to determine whether our DeepDelta models would adhere to these properties. For Eq. 1, we determined the MAE from 0 when DeepDelta predicted the change for pairs of the same molecule. For Eq. 2, we plotted predictions for all molecule pairs against the prediction of those pairs with their order reversed and determine their correlation (Pearson’s r). For Eq. 3, we determined the MAE from 0 for the additivity of predicted differences for all possible groupings of three molecules. Gratifyingly, we observed that the DeepDelta models accurately captured these properties with overall low MAE (0.127 ± 0.042) for the same molecule predictions (Eq. 1), strong anti-correlation (r = − 0.947 ± 0.044) for predictions with swapped inputs (Eq. 2), and overall low MAE (0.127 ± 0.043) for the additive differences (Eq. 3) (Additional file 1: Table S2). Notably, for same molecule predictions (Eq. 1) and additive differences (Eq. 3), the average MAE was over 4 times lower than cross-validation MAE — indicating that DeepDelta can learn these invariants more effectively than it can learn property differences between molecules. Taken together, DeepDelta was able to accurately capture all three properties indicating it was able to learn basic principles of molecular changes.

### Anticipating model performance

**Fig. 4:**
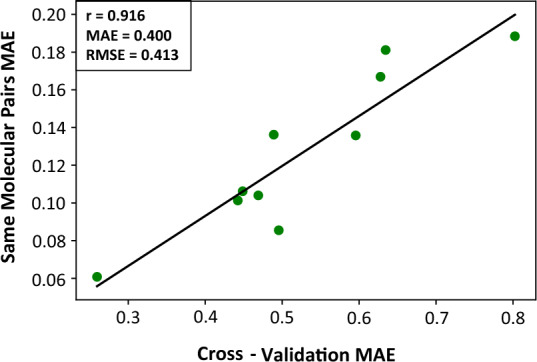
Same molecule MAE correlates with model quality. Correlation plot shows the relationship between the performance of the DeepDelta model on the 10 benchmarking datasets (*x*-axis) and the ability of the DeepDelta models trained on these data to correctly predict same molecular pairs to have no change in property values (*y*-axis, Eq. 1)

Although DeepDelta models trained on different datasets were overall compliant with the three properties of interest (i.e., Eqs. 1–3), the performance of specific DeepDelta models on these mathematically fundamental tasks varied between datasets. We hypothesized that stronger performance on these tasks might correlate with overall performance of the DeepDelta models and thereby provide a measure of model convergence and applicability to a specific dataset. We evaluated whether (1) the MAE of same molecule predictions could predict the MAE of cross-validation performance, (2) the Pearson’s r of the swapped inputs would be inversely correlated to the Pearson’s r of the cross-validation, and (3) the MAE of additive differences would correlate with the MAE of the cross-validations. We found that a model’s ability to correctly predict no change in property between the same molecules correlated strongly (r = 0.916) with overall cross-validation performance (Fig. 4) and that this correlation was consistently stronger than that caused simply by the magnitude of variance found in the values across the datasets (r = 0.746) and was maintained when outlier datasets with variance greater than 1 were removed (Additional file 1: Fig. S4). Additionally, we observed that r values of the swapped inputs were inversely correlated with the r values from cross-validation (r = − 0.729, Additional file 1: Fig. S5) and the MAE values of additive differences were strongly correlated with the MAE from cross-validation (r = 0.918, Additional file 1: Fig. S6). Therefore, these mathematically fundamental calculations are indicative of the stability of the models and their overall performance. As these calculations can be performed on unlabeled data, this approach could serve as an indicator of how well a model will extrapolate to new chemical spaces.

### Predicting large property differences

To further characterize the performance of our DeepDelta models, we next investigated whether the performance on individual predictions correlates with the magnitude of the observed property difference between the two molecules (Additional file 1: Fig. S7, Table S3), i.e., whether it is easier for our models to correctly predict small property changes and harder for the models to accurately predict more drastic property differences. Across all datasets, DeepDelta predictions showed weak correlation between predictive error on individual datapoints and the absolute difference of properties between the two paired molecules (median Pearson’s r of 0.3), but this correlation was stronger for the established ChemProp (median Pearson’s r of 0.5) and Random Forest (median Pearson’s r of 0.6) models. On the level of individual datasets, the correlation for DeepDelta was smaller in 9/10 datasets compared to ChemProp (*p* = 0.01) and in 10/10 datasets compared to Random Forest (*p* = 0.002), indicating that DeepDelta is capable of more accurately predicting larger property changes between two molecules compared to established models in all but one case. To further support this claim, we analyzed the error when predicting only the highest 10% of delta values (i.e., we evaluated only the molecular pairs with the largest difference in property values of the test fold) and observed that DeepDelta exhibited the lowest error for 10/10 datasets compared to Random Forest (*p* = 0.002) and ChemProp (*p* = 0.002). However, DeepDelta did exhibit the highest error rates for predicting the lowest 10% of delta values (*p* = 0.002). This might potentially be driven by the loss function being less affected by errors on small property differences during model training, which could be improved in future model architectures specifically designed to predict small property differences. It is important to also note that these small property value differences lie well within experimental noise and variation and might therefore not be as reliable. Improved experimental resolution and automation should reduce noise and experimental error that may be common within the smallest molecular property deviations. At the same time, we did not observe a strong correlation between the chemical similarity of the molecules and the predictive error (Additional file 1: Fig. S8), and this trend mimicked the distribution between chemical similarity and the ground-truth difference between the paired molecules for the property of interest (Additional file 1: Fig. S9). This data highlights that DeepDelta outperforms established approaches particularly when predicting large property differences between distinct molecules, positioning it for challenging molecular optimization where large property changes are necessary.

### Scaffold-hopping potential

We next tested whether our DeepDelta model could more accurately predict pairs with the same or with different molecular scaffolds. To this end, we separated molecular pairs in the test fold into two groups (pairs with the same scaffold or pairs with different scaffolds) and evaluated the performance of the model trained on the training folds on both groups. DeepDelta predicted properties for pairs with differing Murcko scaffolds with similar accuracy (*p* = 0.11) compared to pairs with the same scaffold (Additional file 1: Fig. S10, Table S4), indicating this method is robust to major structural alterations. Although ChemProp and Random Forest also showed good performance for molecules with differing scaffolds, DeepDelta outperformed both models when predicting molecular pairs with distinct scaffolds with a moderate but significant average improvement of 0.04 in terms of Pearson’s r (*p* = 0.004, Additional file 1: Table S4) and a small, but significant average improvement of 0.04 in terms of MAE (*p* = 0.01, Additional file 1: Table S4). On the level of individual datasets, DeepDelta shows improvement over ChemProp in 8/10 datasets and Random Forest in 9/10 datasets in terms of Pearson’s r, altogether indicating that DeepDelta has potential to guide molecular optimizations that involve scaffold hopping. This better performance at scaffold hopping does not make DeepDelta worse at predicting changes between molecules sharing the same scaffold compared to Random Forest or ChemProp, as DeepDelta showed statistically indistinguishable performance to these models both in terms Pearson’s r (*p* > 0.3) and MAE (*p* > 0.1), meaning DeepDelta presents itself as the model of choice to enable optimization of compounds within the same scaffold as well as to perform scaffold hoping.

## Discussion and conclusion

We here conceived, implemented, validated, and characterized DeepDelta, a novel deep machine learning approach that allows for direct training on and prediction of property differences between two molecules. Given the importance of ADMET property optimization for drug development [34], we here specifically tested our method for 10 established ADMET property benchmarking datasets [18–27]. These are challenging tasks for molecular machine learning given the complexity of the modeled processes, which often involve intricate tissue interactions of molecules, and the small dataset sizes, commonly derived from low-throughput in vivo experiments [35]. Our approach, DeepDelta, outperforms the established, state-of-the-art molecular machine learning models ChemProp and Random Forest for predicting property differences between molecules in the majority of our benchmarks (82% for Pearson’s r and 73% for MAE), including all external test datasets. DeepDelta represents, to the best of our knowledge, the first attempt to directly train machine learning models to predict molecular property differences.

DeepDelta appears particularly powerful when predicting larger property changes (Additional file 1: Fig. S7) and can also predict differences between molecules with different scaffolds more effectively (Additional file 1: Fig. S10), indicating that DeepDelta might be particularly suitable to optimize compounds with drastic ADMET liabilities that might benefit from scaffold hopping into new compound classes. Competitive performance within the same scaffold class indicates that DeepDelta is equally applicable for more fine-grained optimization. DeepDelta benefits from directly learning property difference and data augmentation that increases training datapoints for deep neural networks while also cancelling systematic errors within datasets through pairing. However, pairwise methods like DeepDelta have increased computational costs for model training given the combinatorial expansion of training data sets. As such, we believe these methods are optimally suited for smaller datasets (< 1500 datapoints) and provide the benefit of allowing these smaller datasets to be appropriately applied to data-hungry deep learning models.

Several other molecular pairing approaches have been deployed for various purposes. For example, the pairwise difference regression (PADRE) approach trains machine learning models on pairs of feature vectors to improve the predictions of absolute property values and their uncertainty estimation [36]. PADRE similarly benefits for combinatorial expansion of data; however, PADRE predicts absolute values of unseen molecules like traditional methods instead of being tailored for prediction of property differences. Similarly, Lee and colleagues have used pairwise comparisons to allow for use of qualitative measurements with quantitative ones [37] and AstraZeneca has created workflows that utilize compound pairs to train Siamese neural networks to classify the bioactivity of small molecules [38]. These classification-based methods can allow for direct handling of truncated values through Boolean comparisons. In contrast, the regression-based DeepDelta provides a means of quantifying molecular differences. In computational chemistry, Δ-Machine Learning approaches aim to accelerate and improve quantum property computations by using machine learning to anticipate property differences to a baseline [39]. We believe that existing molecular pairing approaches deployed for other purposes [36–39] will be synergistic with our DeepDelta approach and have the potential to augment or replace standard molecular machine learning approaches for intricate optimization and discovery tasks, especially for complex properties and small datasets.

An intriguing property of DeepDelta is its ability to adhere to mathematical invariants, such as the prediction of zero changes when inputting the same molecule (Eq. 1), the expected inverse relationships when molecule order was inverted (Eq. 2), and the additivity of the predicted differences (Eq. 3) — all of which indicate the models were able to learn basic principles of molecular changes. Interestingly, the performance of the models on these tasks correlated strongly with overall cross-validation performance (Fig. 4), suggesting that such unsupervised calculations could be indicative of model performance and convergence and thereby allow for increased transparency and determination of model applicability to specific datasets. For example, one could evaluate DeepDelta performance on the invariant calculations across a number of new datasets as a predictor of how the DeepDelta approach would likely perform on these datasets to prioritize the datasets on which to apply DeepDelta.

Taken together, we believe that DeepDelta and extensions thereof will provide accurate and easily deployable predictions to steer molecular optimization and compound prioritization. We have here shown its applicability to ADMET property comparison, which is of particular importance to drug development to ensure safety and efficacy of medications but notoriously difficult to predict given the complexity of the involved biological processes and the small datasets resulting from complex in vivo experiments. DeepDelta may effectively guide molecular optimization by informing a project team on the most promising candidates to evaluate next or could be directly integrated into automated, robotic optimization platforms to create safer and more effective drug leads through iterative design. Beyond drug development, we expect DeepDelta to also benefit other tasks in biological and chemical sciences to de-risk material optimization and selection.

### Supplementary Information

**Additional file 1. Fig. S1**: Distribution of Training Datapoints. **Fig. S2**: Epoch Optimization for DeepDelta and ChemProp. **Fig. S3**: LGBMsub Model Performance on External Datasets. **Fig. S4**: Zero Difference Predictions Correlate with Cross-Validation Performance. **Fig. S5**: Consistency in magnitude of predictions when swapping molecule order is inversely correlated with model quality. **Fig. S6**: Error from additivity between three molecules correlates with model quality. **Fig. S7**: Comparison of Error and Property Differences between Paired Datapoints Across Benchmark Datasets. **Fig. S8**: Comparison of Absolute Error and Chemical Similarity Across Benchmark Datasets. **Fig. S9**: Comparison of Property Differences between Paired Datapoints and Chemical Similarity Across Benchmark Datasets. **Fig. S10**: Comparison of Predictive Capacity for Matched and Unmatched Scaffold Pairs Across Benchmark Datasets. . **Table S1**: Parameter Optimizations during 5 × 10-Fold Cross-Validation of LGBM Traditional, and LightGBM Delta. **Table S2**: Evaluations of DeepDelta Models on Mathematical Invariants. **Table S3**: Correlation (Pearson’s r) of error and Property Differences between Paired Datapoints following 5 × 10-Fold Cross-Validation Analysis. **Table S4**: Evaluations of 10-Fold Cross-Validation of all Models for Matched and Unmatched Scaffold Pairs.

## Data Availability

The source code, datasets, and results supporting the conclusions of this article are available in the GitHub repository, https://github.com/RekerLab/DeepDelta.

## Abbreviations

ADMET: Absorption, distribution, metabolism, excretion, and toxicity
D-MPNN: Directed-message passing neural network
FEP: Free energy perturbations
LightGBM: Light gradient boosting machine
MMP: Matched molecular pair
MAE: Mean absolute error
PADRE: Pairwise difference regression
RMSE: Root mean squared error
