# Supplementary material for: DeepDelta: predicting ADMET improvements of molecular derivatives with deep learning
Source: J Cheminform. 2023 Oct 27;15:101. doi: 10.1186/s13321-023-00769-x (PMC10605784; doi:10.1186/s13321-023-00769-x)
Supplement: Supplementary file 1 — Additional file 1. Fig. S1: Distribution of Training Datapoints. Fig. S2: Epoch Optimization for DeepDelta and ChemProp. Fig. S3: LGBMsub Model Performance on External Datasets. Fig. S4: Zero Difference Predictions Correlate with Cross-Validation Performance. Fig. S5: Consistency in magnitude of predictions when swapping molecule order is inversely correlated with model quality. Fig. S6: Error from additivity between three molecules correlates with model quality. Fig. S7: Comparison of Error and Property Differences between Paired Datapoints Across Benchmark Datasets. Fig. S8: Comparison of Absolute Error and Chemical Similarity Across Benchmark Datasets. Fig. S9: Comparison of Property Differences between Paired Datapoints and Chemical Similarity Across Benchmark Datasets. Fig. S10: Comparison of Predictive Capacity for Matched and Unmatched Scaffold Pairs Across Benchmark Datasets. . Table S1: Parameter Optimizations during 5 × 10-Fold Cross-Validation of LGBM Traditional, and LightGBM Delta. Table S2: Evaluations of DeepDelta Models on Mathematical Invariants. Table S3: Correlation (Pearson’s r) of error and Property Differences between Paired Datapoints following 5 × 10-Fold Cross-Validation Analysis. Table S4: Evaluations of 10-Fold Cross-Validation of all Models for Matched and Unmatched Scaffold Pairs. [file 13321_2023_769_MOESM1_ESM.docx]

Supporting Information

DeepDelta: Predicting ADMET Improvements of Molecular Derivatives with Deep Learning

Zachary Fralish^1^, Ashley Chen^2^, Paul Skaluba^1^, & Daniel Reker^1*^

^1^ *Department of Biomedical Engineering, Duke University, Durham, NC 27708, USA*

^2^ *Department of Computer Science, Duke University, Durham, NC 27708, USA*

* Corresponding Author: Daniel Reker, [daniel.reker@duke.edu](mailto:daniel.reker@duke.edu)

**Supplementary Figures**

**
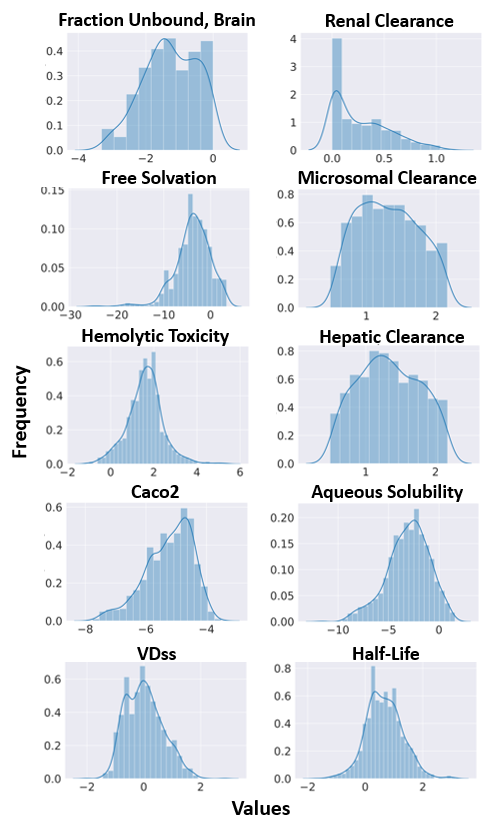
**

**Supplementary Figure 1: Distribution of Training Datapoints.** Distribution of training datapoints across benchmarking datasets. Units are as follows: Fraction Unbound in Brain, Log(f_u,brain_); Renal Clearance, Log(CLr); Free Solvation, Experimental Hydration Free Energy in Water; Microsomal Clearance, Log(mL/min/kg cleared); Hemolytic Toxicity, Log(HD_50_); Hepatic Clearance, Log(mL/min/kg cleared); Caco2, Log(Papp); Aqueous Solubility, LogS; Volume of Distribution at Steady State (VDss), Log(Body/Blood Concentration in L/kg); Half-Life, Log(Half-Life in Hours).


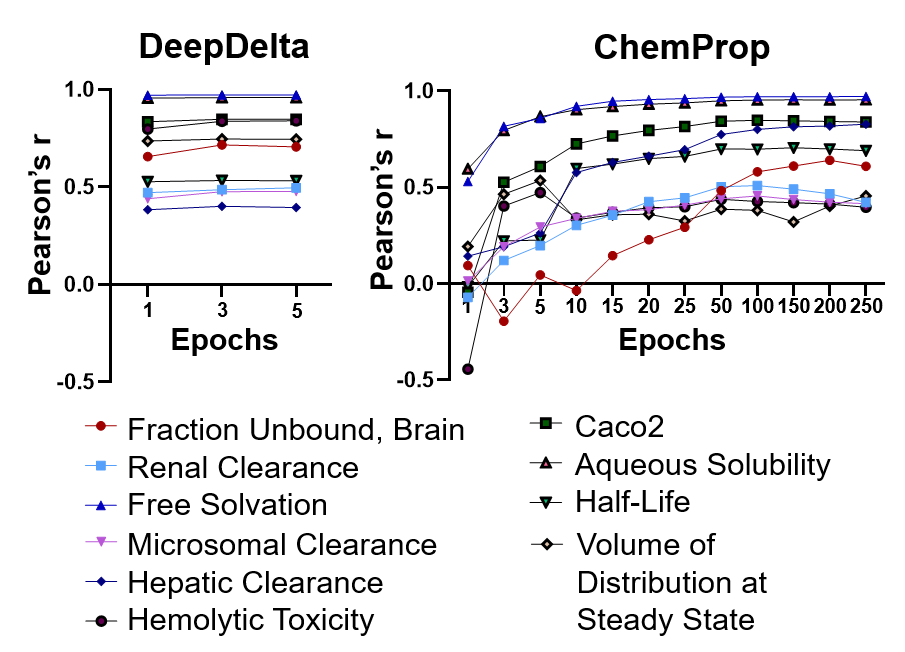


**Supplementary Figure 2: Epoch Optimization for DeepDelta and ChemProp.** Plots of model performance in terms of Pearson’s r across epochs for DeepDelta (left) and ChemProp (right). Selected values were epochs = 5 for DeepDelta and epochs = 50 for ChemProp.

**
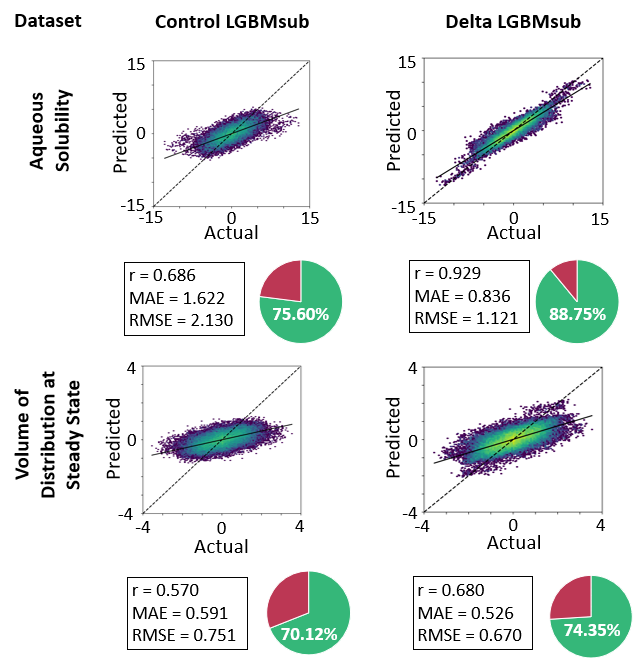
**

**Supplementary Figure 3: LGBMsub** **Model Performance on External Datasets.** Correlation plots, Pearson’s r values, MAE, RMSE, and total percent of predictions correctly indicating a positive or negative change from the starting molecule for control and delta LGBMsub models on cross-merged external test sets. Aqueous solubility is in units of logS and volume of distribution at steady state is in units of log(body/blood concentration in L/kg). Coloring is based on data density with the most densely populated regions shown in yellow, medium density shown in green, and least dense regions in blue and linear interpolation between these groups.


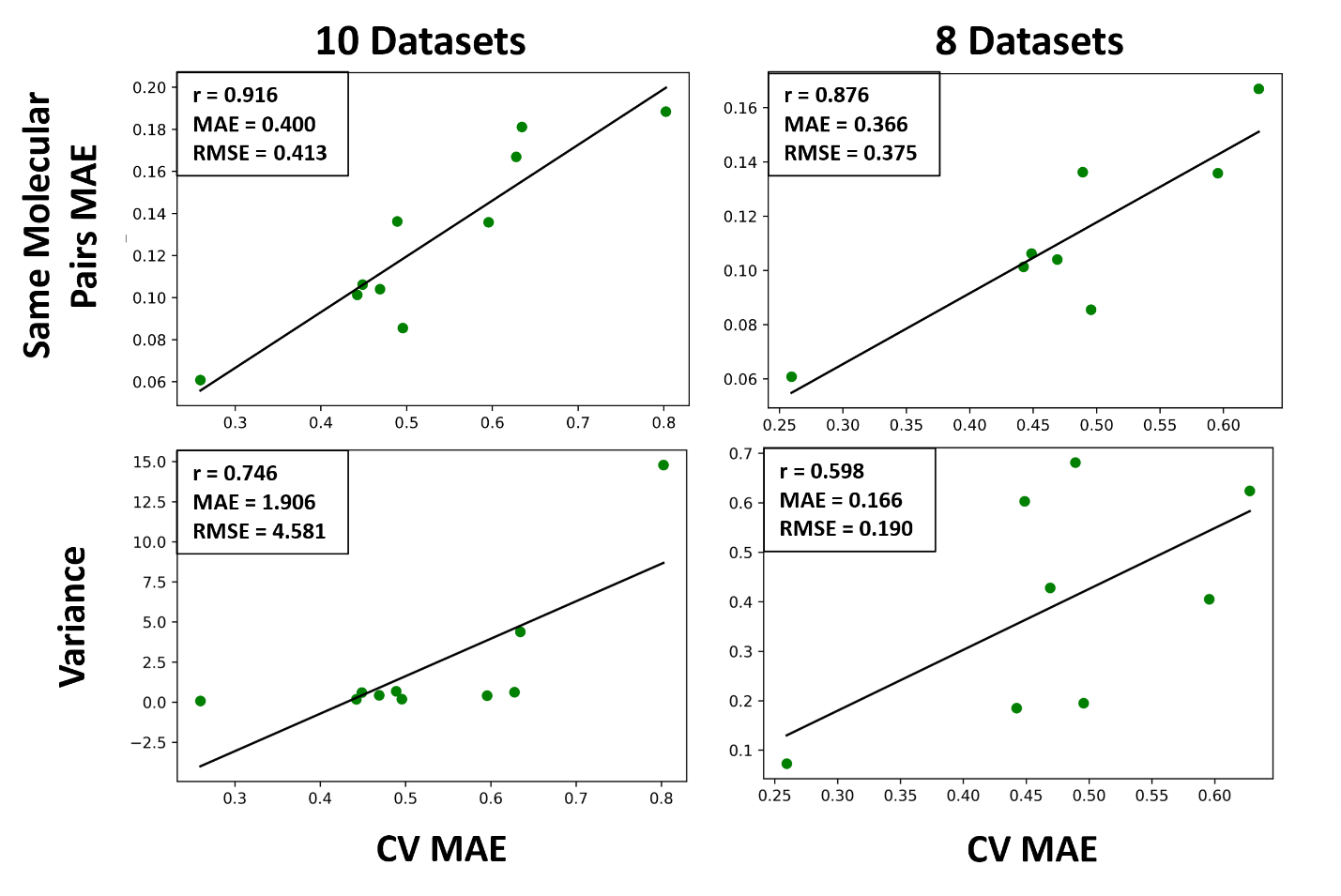


**Supplementary Figure 4: Zero Difference Predictions Correlate with Cross-Validation Performance.** Plots correlating the MAE of the zero difference predictions (eq. 1) with the MAE of the cross-validation plots show higher rates of correlation (top) than plots of the inherent variance of the original dataset with the MAE of the cross-validation (bottom). This trend is maintained when outlier datasets with large variance greater than 1 are removed (eight datasets remaining, right).


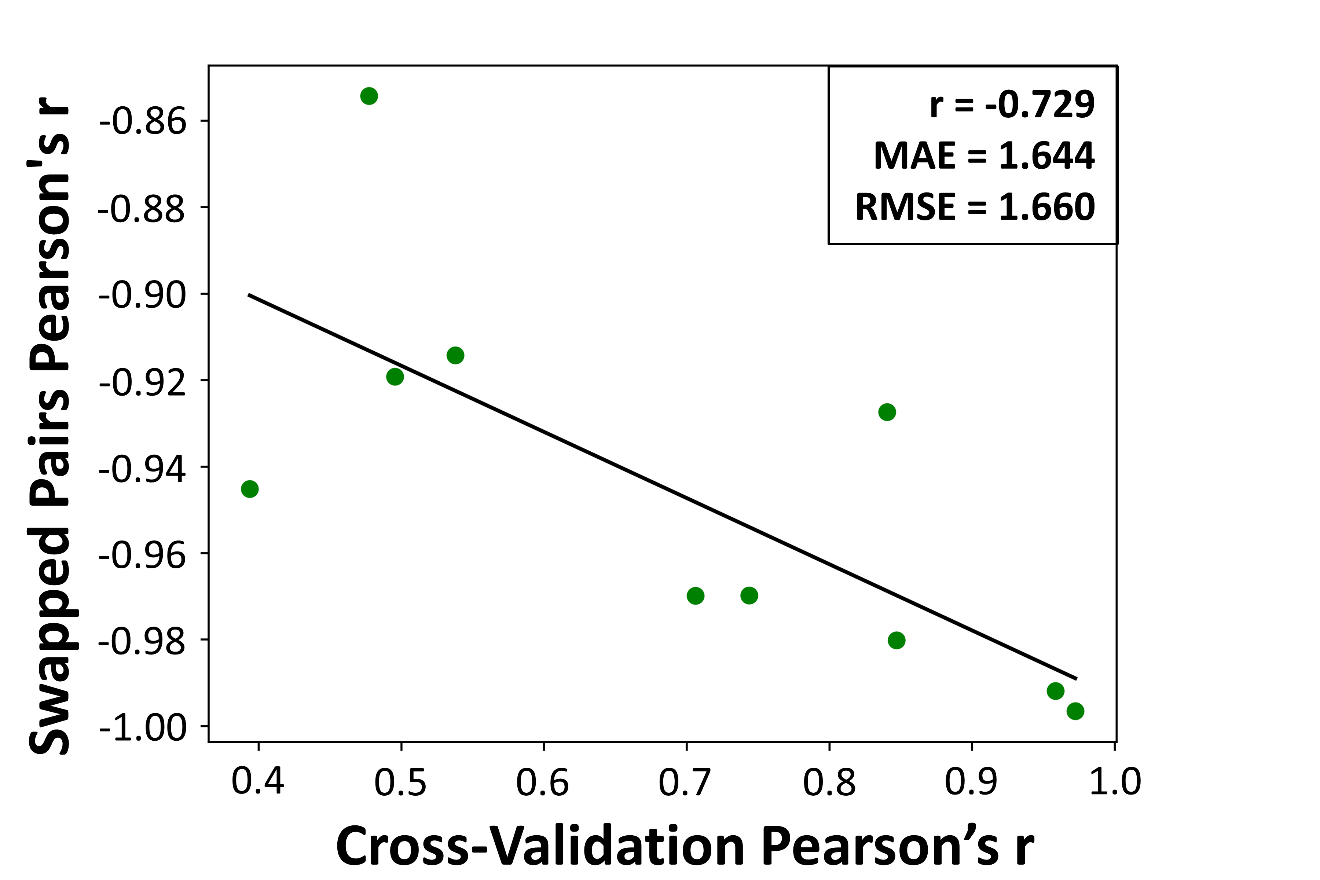


**Supplementary Figure 5: Consistency in magnitude of predictions when swapping molecule order is inversely correlated with model quality.** Correlation plot shows the relationship between the performance of the DeepDelta model on the 10 benchmarking datasets (x axis) with the ability of the DeepDelta models trained on these data to correctly predict swapped molecular pairs to have inverted property values (y axis, Eq. 2).


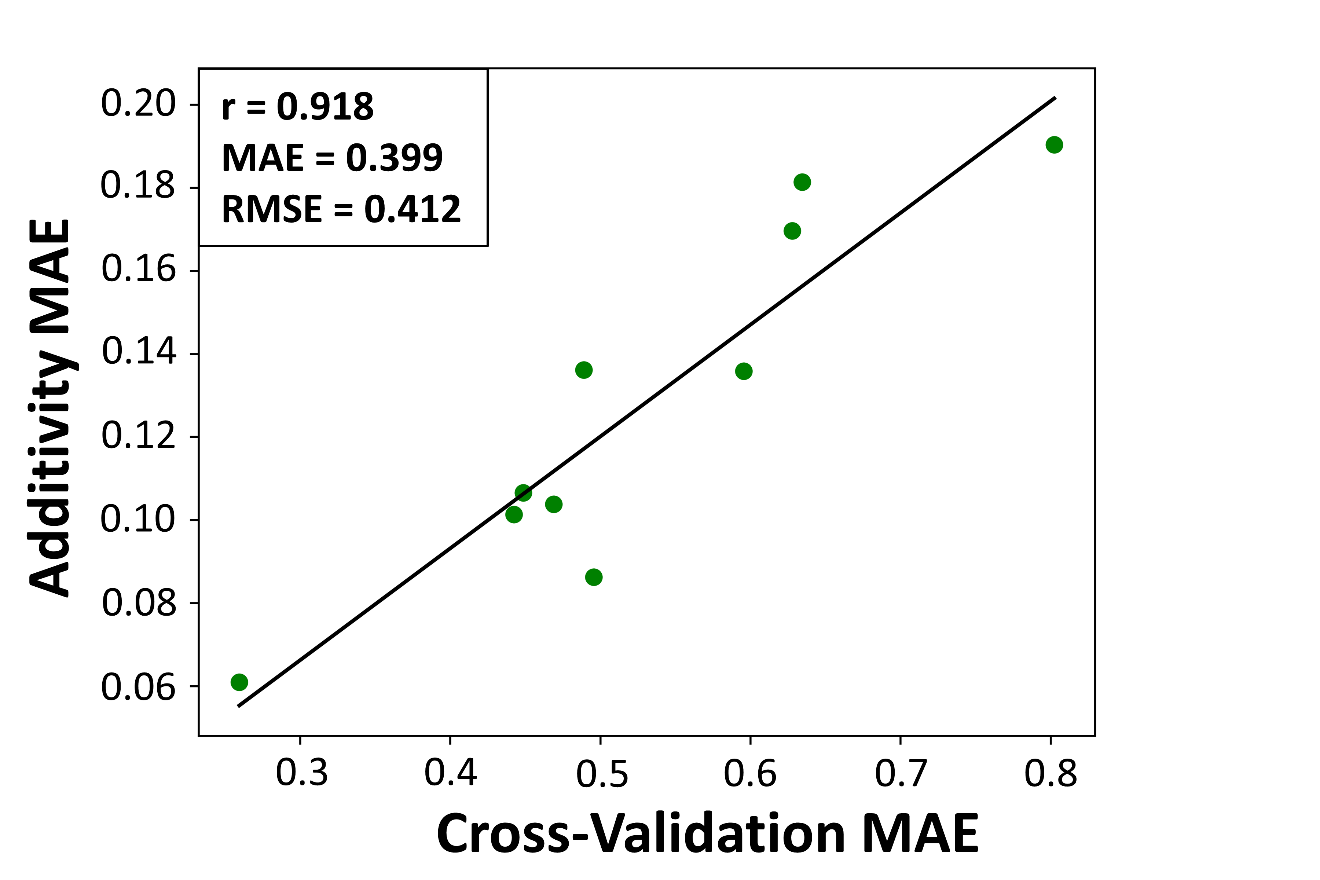


**Supplementary Figure 6: Error from additivity between three molecules correlates with model quality.** Correlation plot shows the relationship between the performance of the DeepDelta model on the 10 benchmarking datasets (x axis) with the ability of the DeepDelta models trained on these data to correctly preserve additivity for predicted differences between three molecules (y axis, Eq. 3).


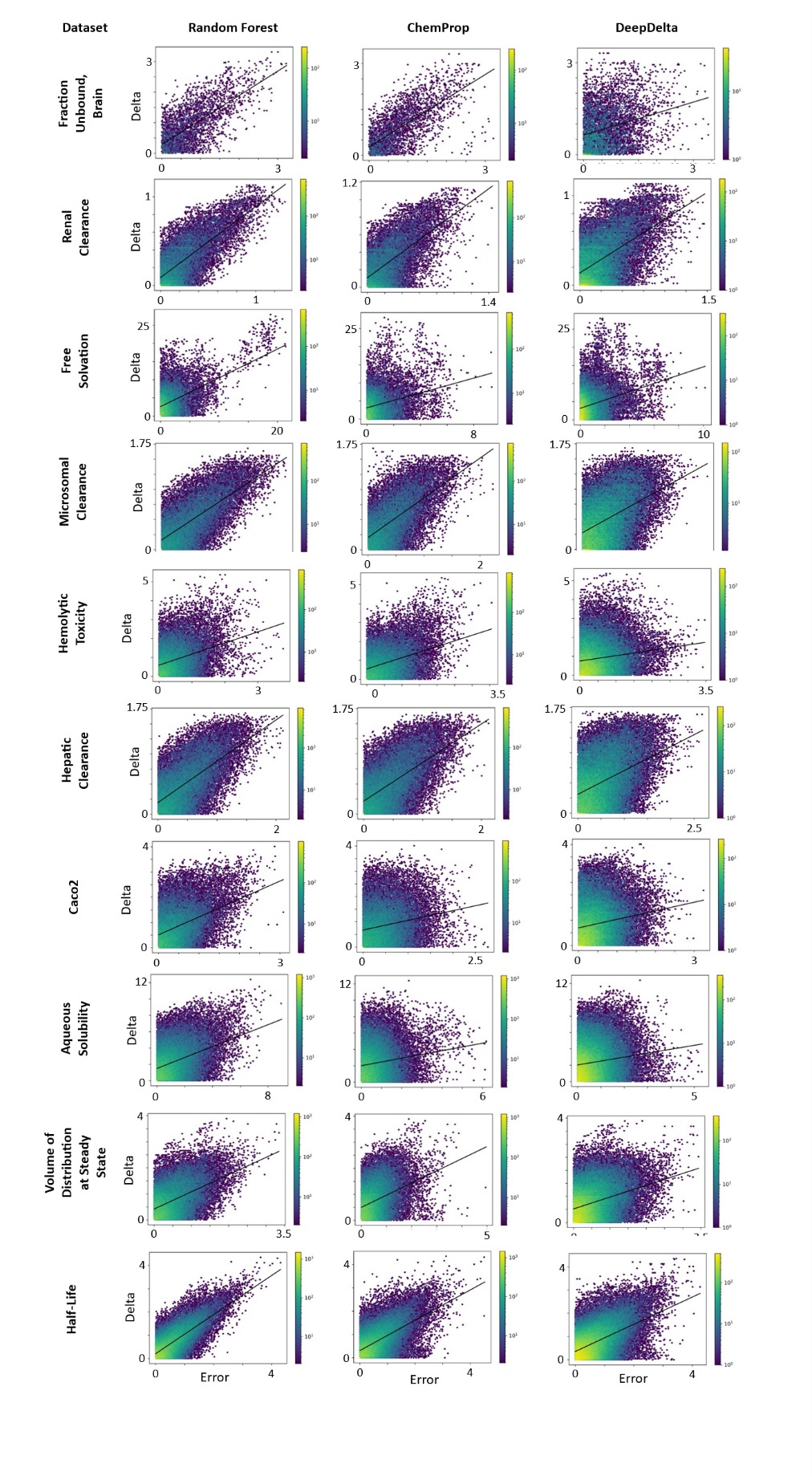


**Supplementary Figure 7: Comparison of Error and Property Differences between Paired Datapoints Across Benchmark Datasets.** Correlation plots for Random Forest (left), ChemProp (middle), and DeepDelta (right) following 5x10-fold cross-validation. Delta represents the difference in value between paired datapoints for the property of interest. Units are as follows: Fraction Unbound in Brain, Log(f_u,brain_); Renal Clearance, Log(CLr); Free Solvation, Experimental Hydration Free Energy in Water; Microsomal Clearance, Log(mL/min/kg cleared); Hemolytic Toxicity, Log(HD_50_); Hepatic Clearance, Log(mL/min/kg cleared); Caco2, Log(Papp); Aqueous Solubility, LogS; Volume of Distribution at Steady State, Log(Body/Blood Concentration in L/kg); Half-Life, Log(Half-Life in Hours).

**
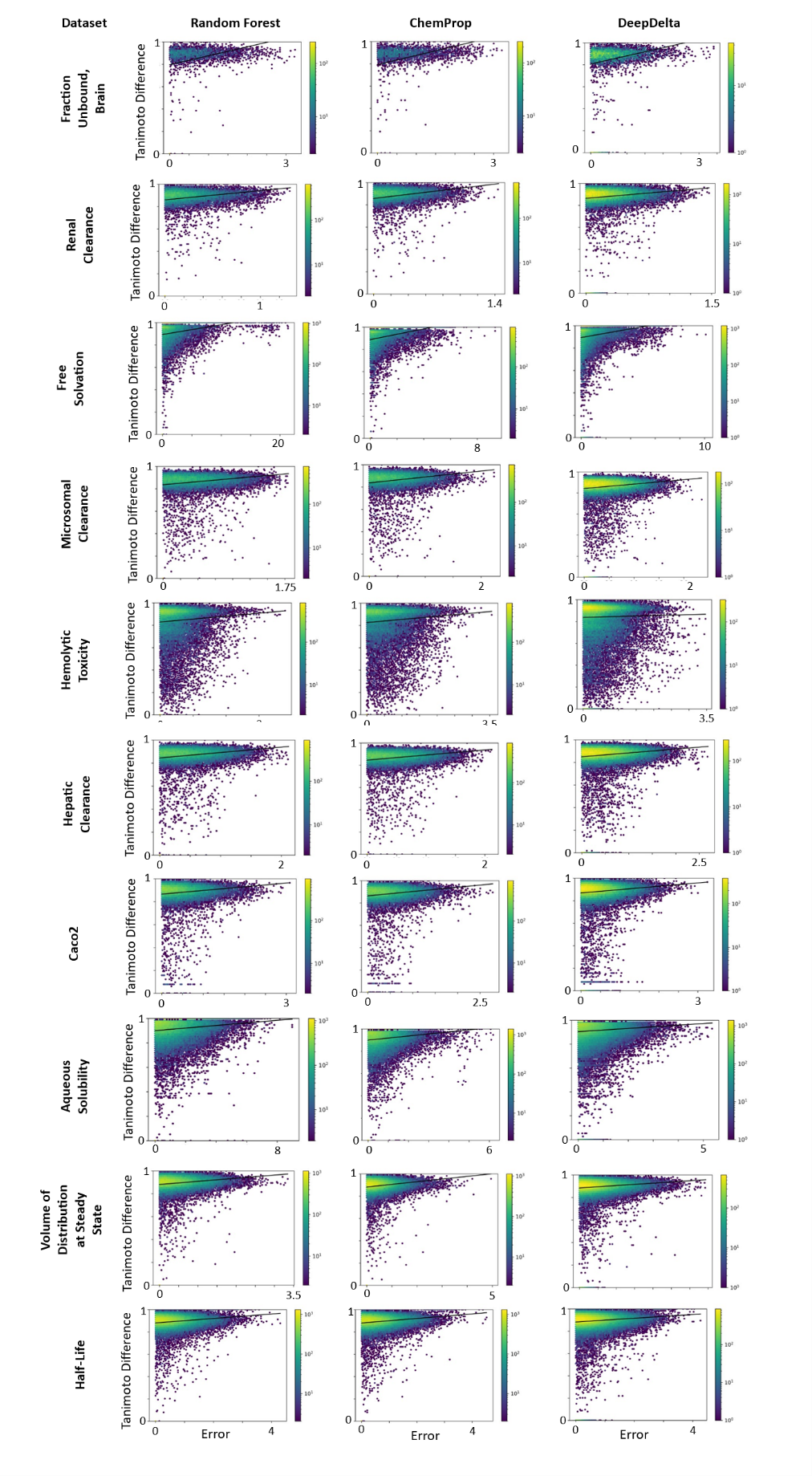
**

**Supplementary Figure 8: Comparison of Absolute Error and Chemical Similarity Across Benchmark Datasets.** Correlation plots for Random Forest (left), ChemProp (middle), and DeepDelta (right) following 5x10-fold cross-validation. Delta represents the difference in values between paired datapoints for the property of interest, and the Tanimoto similarity is a measure of the similarity between the two paired structures, where a value of 1 indicates maximum similarity and 0 indicates no similarity. Units are as follows: Fraction Unbound in Brain, Log(f_u,brain_); Renal Clearance, Log(CLr); Free Solvation, Experimental Hydration Free Energy in Water; Microsomal Clearance, Log(mL/min/kg cleared); Hemolytic Toxicity, Log(HD_50_); Hepatic Clearance, Log(mL/min/kg cleared); Caco2, Log(Papp); Aqueous Solubility, LogS; Volume of Distribution at Steady State, Log(Body/Blood Concentration in L/kg); Half-Life, Log(Half-Life in Hours).

**
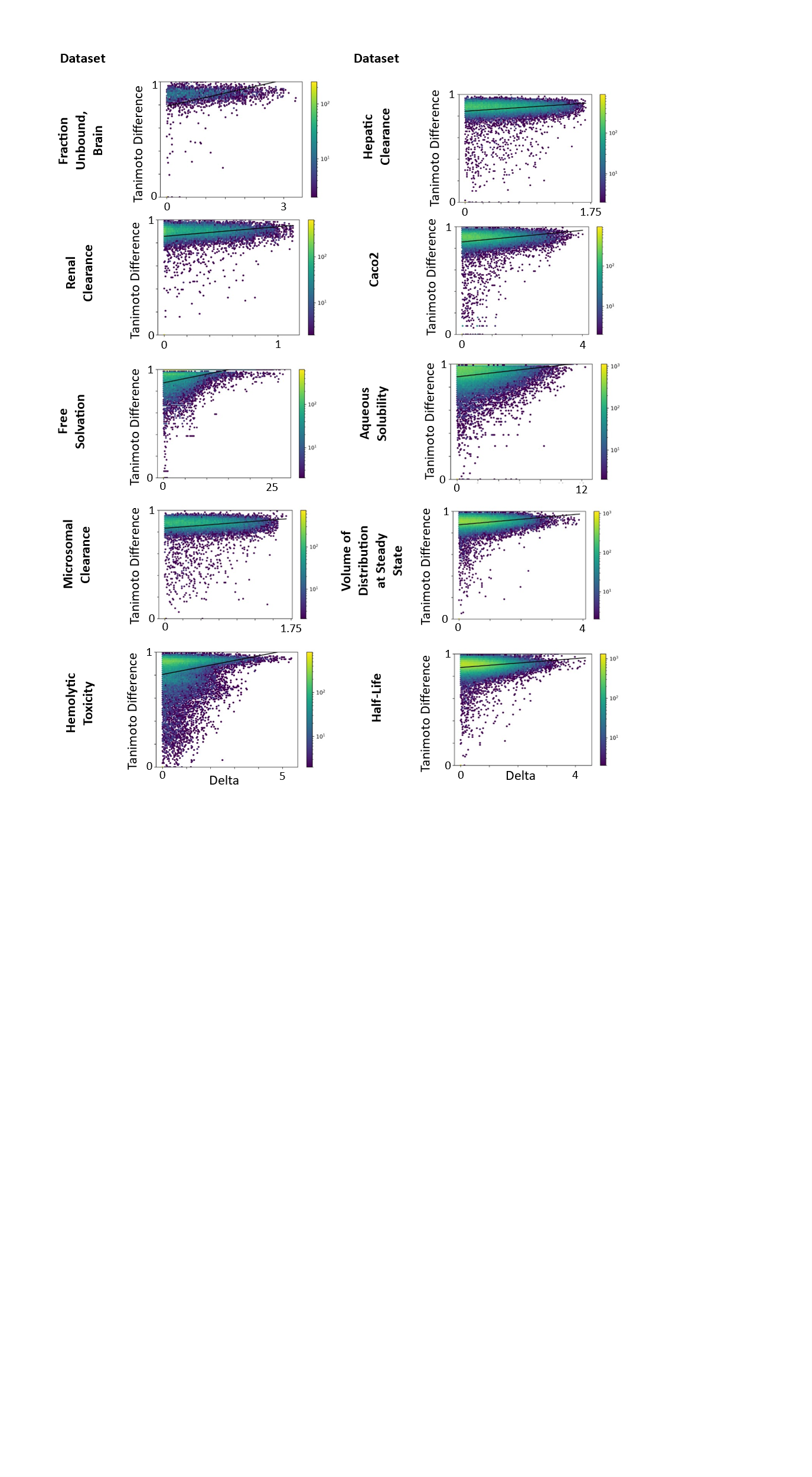
**

**Supplementary Figure 9: Comparison of Property Differences between Paired Datapoints and Chemical Similarity Across Benchmark Datasets.** Correlation plots for Random Forest, ChemProp, and DeepDelta following 5x10-fold cross-validation. Delta represents the difference in value between paired datapoints for the property of interest. Units are as follows: Fraction Unbound in Brain, Log(f_u,brain_); Renal Clearance, Log(CLr); Free Solvation, Experimental Hydration Free Energy in Water; Microsomal Clearance, Log(mL/min/kg cleared); Hemolytic Toxicity, Log(HD_50_); Hepatic Clearance, Log(mL/min/kg cleared); Caco2, Log(Papp); Aqueous Solubility, LogS; Volume of Distribution at Steady State, Log(Body/Blood Concentration in L/kg); Half-Life, Log(Half-Life in Hours).


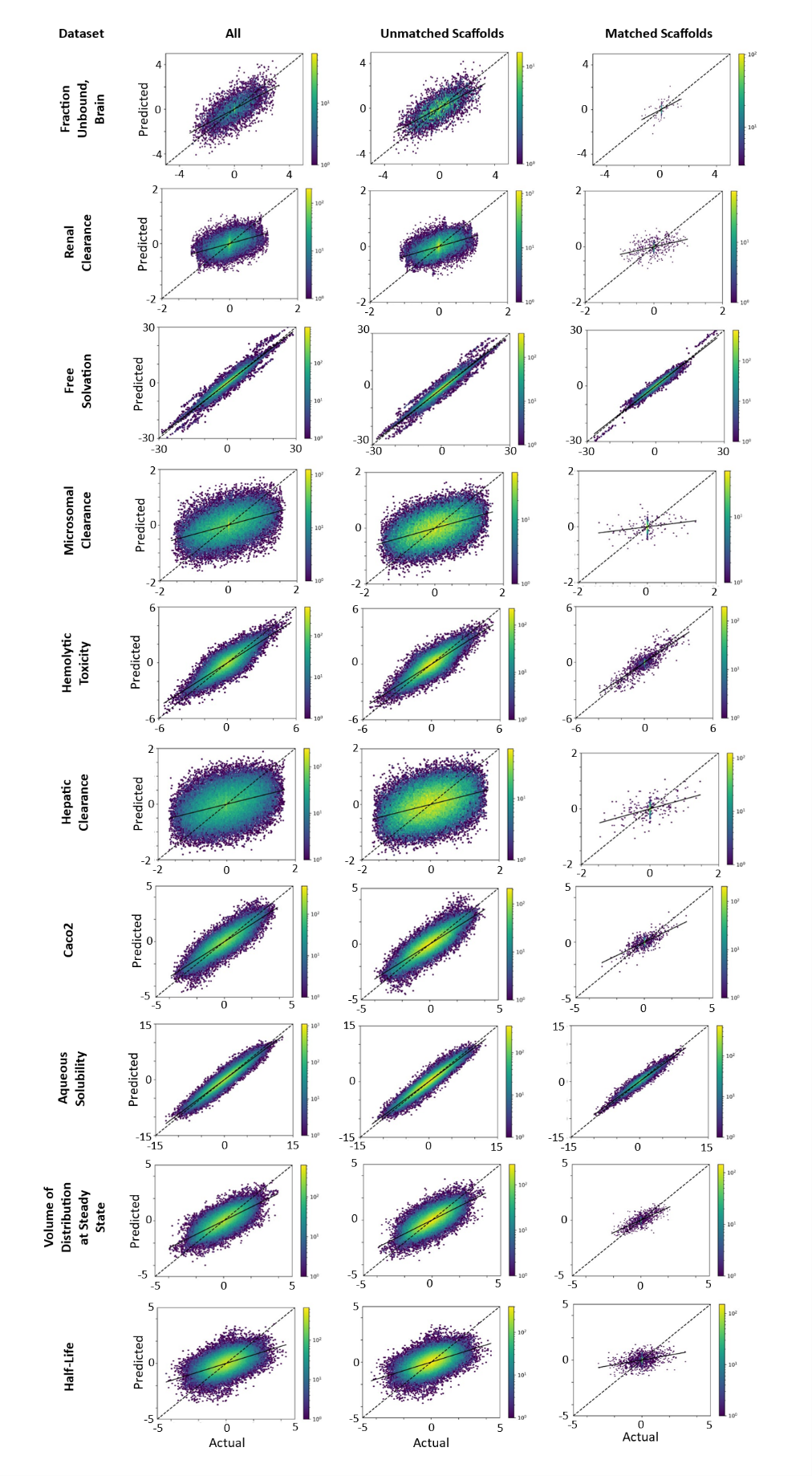


**Supplementary Figure 10: Comparison of Predictive Capacity for Matched and Unmatched Scaffold Pairs Across Benchmark Datasets.** Correlation plots for predictions by DeepDelta for all datapoints, unmatched scaffolds, and matched scaffolds following 5x10-fold cross-validation. Units are as follows: Fraction Unbound in Brain, Log(f_u,brain_); Renal Clearance, Log(CLr); Free Solvation, Experimental Hydration Free Energy in Water; Microsomal Clearance, Log(mL/min/kg cleared); Hemolytic Toxicity, Log(HD_50_); Hepatic Clearance, Log(mL/min/kg cleared); Caco2, Log(Papp); Aqueous Solubility, LogS; Volume of Distribution at Steady State, Log(Body/Blood Concentration in L/kg); Half-Life, Log(Half-Life in Hours). We note that, although the scaffold matching leads to smaller datasets compared to unmatched scaffolds, this matching was only done in the test-fold and not the training-fold. Therefore, the same model is compared in all comparisons and model performance is not impacted by the size of the datasets created through matching scaffolds.

**Supplementary Tables**

**Supplementary Table 1: Parameter Optimizations during 5x10-Fold Cross-Validation of LGBM Traditional, and LightGBM Delta.** Average and standard deviation of Pearson’s r, mean absolute error (MAE), and root mean squared error (RMSE) are presented for all 3 models.

|  | **Pearson’s r** | | | **MAE** | | | **RMSE** | | |
| --- | --- | --- | --- | --- | --- | --- | --- | --- | --- |
| Dataset | LGBM Trad | LGBM Trad | LGBM Delta | LGBM Trad | LGBM Trad | LGBM Delta | LGBM Trad | LGBM Trad | LGBM Delta |
| Subsample Frequency | 0.1 | 1 | 0.1 | 0.1 | 1 | 0.1 | 0.1 | 1 | 0.1 |
| Fraction Unbound, Brain | 0.47 ±0.041 | 0.553 ±0.02 | 0.53 ±0.015 | 1.003 ±0.008 | 0.941 ±0.016 | 0.726 ±0.011 | 1.311 ±0.014 | 1.231 ±0.024 | 0.939 ±0.014 |
| Renal Clearance | 0.283 ±0.016 | 0.453 ±0.011 | 0.443 ±0.007 | 0.307 ±0.003 | 0.36 ±0.004 | 0.265 ±0.002 | 0.396 ±0.004 | 0.458 ±0.005 | 0.344 ±0.002 |
| Free Solvation | 0.589 ±0.007 | 0.83 ±0.005 | 0.898 ±0.003 | 4.23 ±0.058 | 4.283 ±0.034 | 1.656 ±0.027 | 5.472 ±0.064 | 5.506 ±0.067 | 2.415 ±0.037 |
| Microsomal Clearance | 0.273 ±0.011 | 0.409 ±0.007 | 0.42 ±0.01 | 0.509 ±0.004 | 0.581 ±0.004 | 0.443 ±0.002 | 0.638 ±0.005 | 0.732 ±0.004 | 0.556 ±0.003 |
| Hemolytic Toxicity | 0.706 ±0.007 | 0.832 ±0.003 | 0.816 ±0.003 | 0.897 ±0.008 | 0.956 ±0.004 | 0.516 ±0.004 | 1.127 ±0.01 | 1.231 ±0.003 | 0.672 ±0.005 |
| Hepatic Clearance | 0.28 ±0.013 | 0.418 ±0.005 | 0.424 ±0.013 | 0.529 ±0.006 | 0.625 ±0.004 | 0.46 ±0.005 | 0.663 ±0.008 | 0.786 ±0.005 | 0.575 ±0.005 |
| Caco2 | 0.565 ±0.013 | 0.827 ±0.006 | 0.829 ±0.004 | 0.82 ±0.012 | 0.881 ±0.009 | 0.473 ±0.005 | 1.036 ±0.014 | 1.118 ±0.013 | 0.61 ±0.007 |
| Aqueous Solubility | 0.596 ±0.006 | 0.85 ±0.002 | 0.852 ±0.003 | 2.184 ±0.029 | 2.397 ±0.019 | 1.197 ±0.004 | 2.783 ±0.038 | 3.02 ±0.022 | 1.562 ±0.011 |
| Volume of Distribution at Steady State | 0.578 ±0.006 | 0.73 ±0.004 | 0.719 ±0.003 | 0.772 ±0.006 | 0.778 ±0.004 | 0.493 ±0.003 | 0.969 ±0.008 | 0.984 ±0.006 | 0.64 ±0.003 |
| Half-life | 0.330 ±0.007 | 0.516 ±0.011 | 0.514 ±0.003 | 0.662 ±0.003 | 0.598 ±0.006 | 0.594 ±0.005 | 0.849 ±0.003 | 0.776 ±0.006 | 0.771 ±0.002 |

**Supplementary Table 2: Evaluations of DeepDelta** **Models on Mathematical Invariants.** Mathematical invariants are compared against cross-validation results (with random state = 1) and analyzed with Pearson’s r (r), mean absolute error (MAE), and root mean squared error (RMSE).

|  | **Cross-Validation** | | | **Eq. 1** | **Eq. 2** | **Eq. 3** |
| --- | --- | --- | --- | --- | --- | --- |
| **Dataset** | **r** | **MAE** | **RMSE** | **MAE** | **r** | **MAE** |
| **Fraction Unbound, Brain** | 0.7061 | 0.6278 | 0.812 | 0.1669 | -0.9699 | 0.1696 |
| **Renal Clearance** | 0.4955 | 0.2595 | 0.339 | 0.0608 | -0.9192 | 0.0609 |
| **Free Solvation** | 0.9724 | 0.8024 | 1.271 | 0.1884 | -0.9966 | 0.1904 |
| **Microsomal Clearance** | 0.4774 | 0.4424 | 0.5544 | 0.1013 | -0.8543 | 0.1013 |
| **Hemolytic Toxicity** | 0.8404 | 0.4891 | 0.6411 | 0.1362 | -0.9274 | 0.1361 |
| **Hepatic Clearance** | 0.3938 | 0.4956 | 0.6234 | 0.0855 | -0.9452 | 0.0862 |
| **Caco2** | 0.8471 | 0.4487 | 0.5797 | 0.1062 | -0.9802 | 0.1065 |
| **Solubility** | 0.9585 | 0.6345 | 0.843 | 0.1811 | -0.9919 | 0.1814 |
| **Volume of Distribution at Steady State** | 0.7438 | 0.469 | 0.6207 | 0.104 | -0.9698 | 0.1038 |
| **Half-life** | 0.5378 | 0.5956 | 0.7726 | 0.1358 | -0.9143 | 0.1358 |

**Supplementary Table 3: Correlation (Pearson’s r) of error and Property Differences between Paired Datapoints following 5x10-Fold Cross-Validation Analysis.**

| **Dataset** | **Random Forest** | **ChemProp** | **DeepDelta** |
| --- | --- | --- | --- |
| **Fraction Unbound, Brain** | 0.698 | 0.707 | 0.274 |
| **Renal Clearance** | 0.731 | 0.634 | 0.544 |
| **Free Solvation** | 0.505 | 0.285 | 0.314 |
| **Microsomal Clearance** | 0.699 | 0.617 | 0.456 |
| **Hemolytic Toxicity** | 0.343 | 0.367 | 0.156 |
| **Hepatic Clearance** | 0.642 | 0.601 | 0.411 |
| **Caco2** | 0.419 | 0.207 | 0.185 |
| **Aqueous Solubility** | 0.388 | 0.154 | 0.146 |
| **Volume of Distribution at Steady State** | 0.480 | 0.368 | 0.330 |
| **Half-life** | 0.736 | 0.591 | 0.516 |

**Supplementary Table 4: Evaluations of 10-Fold Cross-Validation of all Models for Matched and Unmatched Scaffold Pairs.** Pearson’s r, mean absolute error (MAE), and root mean squared error (RMSE) are listed as the 1^st^, 2^nd^, and 3^rd^ number in each cell, respectively (random state = 1).

|  | **DeepDelta** | | **ChemProp** | | **Random Forest** | |
| --- | --- | --- | --- | --- | --- | --- |
| **Dataset** | **Matched Scaffolds** | **Unmatched Scaffolds** | **Matched Scaffolds** | **Unmatched Scaffolds** | **Matched Scaffolds** | **Unmatched Scaffolds** |
| **Fraction Unbound, Brain** | 0.455  0.228  0.339 | 0.707  0.648  0.828 | 0.142  0.095  0.294 | 0.488  0.770  0.977 | 0.110  0.092  0.294 | 0.533  0.744  0.946 |
| **Renal Clearance** | 0.388  0.142  0.227 | 0.497  0.262  0.341 | 0.434  0.098  0.209 | 0.504  0.259  0.333 | 0.485  0.093  0.203 | 0.466  0.257  0.338 |
| **Free Solvation** | 0.979  0.566  0.902 | 0.971  0.912  1.409 | 0.971  0.676  1.057 | 0.967  1.051  1.487 | 0.792  1.698  2.719 | 0.852  1.974  3.061 |
| **Microsomal Clearance** | 0.201  0.142  0.232 | 0.478  0.447  0.558 | 0.319  0.054  0.195 | 0.440  0.446  0.556 | 0.195  0.058  0.201 | 0.444  0.442  0.549 |
| **Hemolytic Toxicity** | 0.848  0.326  0.482 | 0.840  0.494  0.645 | 0.666  0.379  0.657 | 0.776  0.580  0.741 | 0.763  0.320  0.570 | 0.818  0.513  0.673 |
| **Hepatic Clearance** | 0.424  0.130  0.223 | 0.394  0.501  0.627 | 0.057  0.076  0.237 | 0.438  0.459  0.571 | 0.455  0.065  0.202 | 0.430  0.459  0.571 |
| **Caco2** | 0.693  0.222  0.339 | 0.848  0.453  0.583 | 0.773  0.149  0.290 | 0.844  0.465  0.588 | 0.696  0.155  0.329 | 0.824  0.480  0.622 |
| **Aqueous Solubility** | 0.961  0.471  0.644 | 0.958  0.661  0.871 | 0.950  0.511  0.730 | 0.950  0.721  0.954 | 0.792  1.036  1.421 | 0.841  1.269  1.656 |
| **Volume of Distribution at Steady State** | 0.721  0.230  0.350 | 0.744  0.473  0.624 | 0.749  0.174  0.334 | 0.699  0.507  0.671 | 0.763  0.170  0.326 | 0.733  0.485  0.631 |
| **Half-life** | 0.386  0.361  0.569 | 0.533  0.598  0.782 | 0.368  0.281  0.555 | 0.503  0.609  0.792 | 0.474  0.264  0.515 | 0.528  0.590  0.765 |
